# Synthesis and Diuretic Activity of Substituted 1,3,4-Thiadiazoles

**DOI:** 10.1155/2022/3011531

**Published:** 2022-04-08

**Authors:** Asrat Ergena, Yerra Rajeshwar, Gebremedhin Solomon

**Affiliations:** ^1^Department of Pharmaceutical Chemistry, College of Medicine and Health Sciences, University of Gondar, Gondar, Ethiopia; ^2^Department of Medicinal Chemistry, College of Health Sciences, Mekelle University, Mekelle, Ethiopia

## Abstract

1,3,4-Thiadiazole nuclease, a 5-membered heterocyclic ring system containing two nitrogen and one sulfur atoms in addition to carbon atoms, is compound that showed promising results in the process of searching new diuretic agents. In this study, seven 5- and 2-thioate derivatives of 1, 3, 4-thiadiazoles were synthesized by substitution reaction using acetone as solvent and K_2_CO_3_ as a base. The compounds ware then characterized by using IR and NMR spectroscopy. The diuretic activity of the compounds was evaluated on Swiss albino mice by measuring urine volume, urinary pH, and urinary Na^+^, K^+^, and Cl^−^. The result showed increase in urinary excretion of both water and electrolytes. 5-Methyl-substituted derivatives of 1, 3, 4-thiadiazoles showed significant increase in excretion of both water and electrolytes when they are compared to both negative control and 5-amino-substituted derivatives. The highest diuretic activity (0.82) was recorded for para-nitro-substituted benzene ring at 2-thioate group of 5-methyl-1, 3, 4-thiadiazole, while the least (0.56) was recorded for propanethioate group at 2^nd^ position and amine group at 5^th^ position of 1, 3, 4-thiadiazole. The finding of the present study showed that all the compounds have diuretic activity and 5-methyl derivatives of 1, 3, 4-thiadiazoles exhibited significant diuretic activity.

## 1. Introduction

Imbalances and abnormalities in extracellular fluid volume and electrolyte composition are common clinical problems. Drugs that block the transport of electrolytes in the renal tubules are valuable clinical tools to treat these problems [1]. Diuretics are types of drugs that increase the urine formation and thus remove excess extracellular water from edematous tissue. They support the excretion of urine along with increased rate of excretion of electrolytes [2]. They are commonly indicated in heart and kidney problems such as fluid retention (edema), hypertension, congestive heart failure, liver cirrhosis, epilepsy, glaucoma, altitude sickness, and some kidney diseases. Some diuretics like acetazolamide are also used to alkalinize urine and increase the excretion of acidic substances in cases of overdose or poisoning. Diuretics produce their effect by acting on distinct sites of nephron; however, the inhibition of sodium ions reabsorption along the renal tubules of the kidney is a primary mechanism [2, 3].

Thiadiazoles are five-membered ring heterocyclic compounds containing hydrogen-binding domain, sulfur atom, and two-nitrogen system in their structure [4]. The compounds are being utilized as useful intermediates in medicinal chemistry, and they are characterized as structural units for a number of biologically active molecules [5].

Compounds with 1, 3, 4-thiadiazole nuclease have been investigated so far for their antimicrobial activity [5, 6], anticancer activity [7–12], vasodilator activity [13], anti-helicobacter pylori [4], anticonvulsant activity [14], anti-inflammatory activity [15], antitubercular activity [16], wound healing [2], antiviral activity [16, 17], anti-leishmanial activity [18], trypanocidal (anti-epimastigote) activity [19], carbonic anhydrase inhibitory activity [3, 13], antidiabetic activity [4, 8, 20], and effect on telomerase activation [21]. The molecular targets of 1, 3, 4-thiadiazoles include the enzymes carbonic anhydrase (CA), cyclooxygenase (CO), neutral endopeptidase (NEP), aminopeptidase N (APN), matrix metalloproteinases (MMPs), phosphodiesterases (PDEs), and c-Src/Abl tyrosine kinases [22].

Coming to the diuretic activity of thiadiazoles, different studies have been conducted to screen the effect of the compounds on diuresis. The 2^nd^ and the 5^th^ positions of 1, 3, 4-thiadiazole ring are sites for different substituents to exhibit diuretic activity [4, 20, 23]. The most potent compounds with diuretic activity are belonged to sulfonamide and sulfamate classes. However, some investigations done on compounds other than sulfonamides and sulfamates revealed that compounds such as mercaptans showed that SH– moiety may act as zinc-binding function in design of CAIs [9, 12, 24–28].

The experiments conducted to evaluate the diuretic activity of thiadiazoles showed that the compounds are worthy of mention their diuretic activity [24]. Data analysis of the latest literature has shown that, recently, huge work is being conducted to search biologically active entities among 1, 3, 4-thiadiazole derivatives. These projects gave satisfactory results as a number of 1, 3, 4-thiadiazole derivatives are proven for their different biological activities including diuretic activity [6]. By using this as a good starting point, many scholars have been working on this area by targeting different sites to overcome the problems with current diuretics. This work can also be considered as a part of different activities of searching new compounds with diuretic activity. The objective of this study was to synthesize some 1, 3, 4-thiadiazole derivatives and evaluate in *vivo* diuretic activity of on Swiss albino mice.

## 2. Methods

### 2.1. Synthesis

#### 2.1.1. General Procedure

The reactions were started by available samples. 5-Amino- and 5-methyl-substituted 1, 3, 4-thiadiazole-2-thio acyl derivatives were synthesized independently. 0.01 Mol of each reactant, 0.01 mol of potassium carbonate, and 20 ml of dry acetone were refluxed in 100 ml RBF on oil bath for three hours. The progress of the reaction was monitored by TLC using an appropriate mobile phase. After the reaction was completed, excess of dry acetone was distilled off, and after cooling, the reaction mixture was poured in ice cold water. Then, the product was purified by crystallization and column chromatography. The melting points of the compounds were determined on a Böetius PHMK apparatus (Veb Analytik, Dresden, Germany). The TLC was performed using aluminum plates, precoated with silica gel 60 or 60 F254 (Merck, Munchen, Germany), and visualized by UV light (254 nm, Merck, Darmstadt, Germany). The NMR spectra were recorded on a Varian Gemini 300 and Bruker DRX 400 spectrometer (Varian, Palo Alto, CA, USA) at 25°C. The IR-spectra were recorded (KBr) on a Jasco FT/IR-410 instrument (Jasco, Easton, PA, USA) [22, 23].

### 2.2. Biological Activity

#### 2.2.1. Oral Acute Toxicity Test

Oral acute toxicity of the synthesized compounds was investigated according to OECD, 2008, with slight modifications. Female Swiss albino mice were fasted for 4 hours before administering the test compounds. The test compounds were given orally dissolved in 2% of Tween-80 and administered in sequential dose using 175 mg/kg of each compound as starting dose and 3.2 as dose progression factor. 175 Mg/kg of each test compound was given for respective mouse and observed for signs of toxicity for 48 hours, and then, the next dose 550 mg/kg of each compound was administered for one mouse and again observed for signs of toxicity for 48 hours. After 48 hours of administration, the same dose of the compounds was administered for four mice and the survival of the mice was followed for 14 days. Acute toxicity signs like lacrimation, hair erecting, blinking urination, muscle weakness, sedation and convulsion, reduction in motor activity, diarrhea, coma, and death were checked in test mice.

### 2.3. Diuretic Activity

The animals were divided into nine groups, each with six mice. Group I, a negative control group, was administered with 1 ml/100 gram body weight of 2% Tween-80, and Group II, a positive control group, was administered with furosemide 10 mg/kg body weight [28]. The rest seven groups were administered with 200 mg/kg (the dose selected by pilot experiment) body weight of each of seven synthesized compounds.

The diuretic activity of the compounds was determined following the methods used by Katzung with slight modifications [29]. Each mouse was placed in an individual cage 24 hours prior to commencement of the experiment for adaptation and then fasted overnight with free access to water *ad libitum*. The animals were pretreated with physiological saline (0.9% NaCl) at an oral dose of 0.15 ml/10 g body weight. Each of experimental group was then treated accordingly by oral gavage. Immediately after administration, each mouse was individually placed in a metabolic cage. Urine was then collected and measured at 1, 2, 3, 4, 5, and 24 hours after dosing. The pH of the fresh urine samples of all groups was measured with a calibrated digital pH meter. The urine was then filtered and finally stored at −20°C for electrolyte analysis [30]. Urinary Na^+^, K^+^, and Cl^−^ concentrations of the experimental, control, and standard groups were determined using Ion Selective Electrode analyzer (ISE MLSE02) (MEDSINGLONG, Guangzhou, China). The following three parameters were calculated in order to compare the effects of the synthesized compounds with the negative and positive control groups on urine excretion [31, 32]:

(1) $$\begin{array}{c} \text{Urinary Excretion}=\frac{\text{Total Urinary Output}}{\text{Total Liquid Administered}}\times 100,\\ \text{Diuretic Action}=\frac{\text{Urinary Excretion of Treatment Groups}}{\text{Urinary Excretion of Control Group}},\\ \text{ Diuretic Activity}=\frac{\text{Diuretic Action of Test Drug}}{\text{Diuretic Action of Standard Drug }}. \end{array}$$

## 3. Result and Discussion

### 3.1. Chemistry

**Scheme 1:**
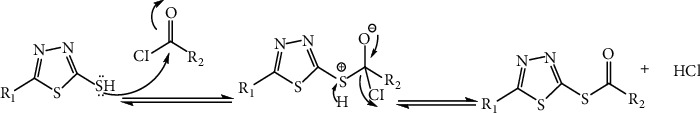
General synthesis mechanism of the test compounds.

In the current study, both 5-amino and 5-methyl-substituted 1, 3, 4-thiadiazole-2-thioacyl derivatives were synthesized independently (Table 1). The reaction mechanism was second-order nucleophilic substitution (S_N_2) reaction (Scheme 1), which involves the nucleophilic attack on partially positive carbon in acyl chlorides by lone pair electrons of sulfur atom from 2-thio-substituted thiadiazoles. Then, the chloride atom from acyl chloride leaves by backward attack from negatively charged oxygen radical and the resulting compounds will be formed. Dry acetone, which is polar aprotic solvent, was used as a solvent, and anhydrous K_2_CO_3_ was used as a base. The reaction was conducted in anhydrous media using anhydrous compounds.

#### 3.1.1. 5-Methyl-1,3,4-Thiadiazol-2-yl Benzothioate (CPD-I)

Yield: 2.03 g (86%); m.p. 134-135; *R*_*f*_: 0.62; IR (KBr, *n*, cm^−1^): 2889 (aliphatic H-C), 1646 (C=N of thiadiazole ring), 743 (S-C of thiadiazole ring), 1710 (C=O group), 1505 (aromatic C-C), 3039 (aromatic C-H); 1H NMR (400 MHz, d, ppm, DMSO-d6): 2.86 ppm (s, 3H, Ar-CH_3_), 7.42–8.03 ppm (d, d, and *t* for Ar-H at ortho-, meta-, and para-position); ^13^C NMR (DMSO-d6): 13 ppm (CH_3_), 153, 162 ppm (thiadiazole), 197 ppm (C=O), 139, 120,102, 126 ppm (aromatic).

#### 3.1.2. 5-Methyl-1,3,4-Thiadiazol-2-yl 4-Methoxybenzothioate (CPD-II)

Yield: 1.81 g (68%); m.p. 175-176; *R*_*f*_: 0.67; IR (KBr, *n*, cm^−1^): 2887 (aliphatic H-C), 1646 (C=N of thiadiazole ring), 743 (S-C of thiadiazole ring), 1712 (C=O group), 1511 (aromatic C-C), 3042 (aromatic C-H), 1140 (methoxy O-C group); 1H NMR (400 MHz, d, ppm, DMSO-d6): 2.86 ppm (s, 3H, Ar-CH_3_), 3.92 ppm (s, 3H, -O-CH_3_), 7.01 ppm (d, 2H, Ar-H), 7.98 ppm (d, 2H, Ar-H); ^13^C NMR (DMSO-d6): 14 ppm (CH_3_), 151, 160 ppm (thiadiazole ring), 193 ppm (C=O) 137, 132, 148, 155 ppm (aromatic), 69 ppm (C-O).

#### 3.1.3. 5-Amino-1,3,4-Thiadiazol-2-yl 4-Nitrobenzothioate (CPD-III)

Yield: 1.78 g (63%); m.p. 246–248; *R*_f_: 0.71; IR (KBr, *n*, cm^−1^): 3325–3112 (N-H), 1668 (C=N of thiadiazole ring), 754 (S-C of thiadiazole ring), 1719 (C=O group), 1532 (aromatic C-C), 3051 (aromatic C-H), 1366–1541 (N-O); 1H NMR: 3.97 ppm (s, 2H, NH_2_), 8.21 ppm (d, 2H, Ar-H), 8.29 ppm (d, 2H, Ar-H); ^13^C NMR (DMSO-d6): 160, 163 ppm (thiadiazole ring), 188 ppm (C=O), 135, 129, 142, 147 ppm (aromatic).

#### 3.1.4. 5-Methyl-1,3,4-Thiadiazol-2-yl 4-Nitrobenzothioate (CPD-IV)

Yield: 2.02 g (72%); m.p. 177-178; *R*_*f*_: 0.58; IR (KBr, *n*, cm^−1^): 2884 (aliphatic H-C), 1661 (C=N of thiadiazole ring), 755 (S-C of thiadiazole ring), 1710 (C=O group), 1536 (aromatic C-C), 3061 (aromatic C-H), 1372–1533 (N=O), 1H NMR (400 MHz, d, ppm, DMSO-d6): 2.50 ppm (s, 3H, Ar-CH_3_), 8.20 ppm (d, 2H, Ar-H), 8.26 ppm (d, 2H, Ar-H); ^13^C NMR (DMSO-d6): 14 ppm (CH_3_), 152, 159 ppm (thiadiazole ring), 187 ppm (C=O), 126, 122, 141, 148 ppm (aromatic).

#### 3.1.5. 5-Methyl-1,3,4-Thiadiazol-2-yl 4-Chlorobenzothioate (CPD-V)

Yield: 1.65 g (61%); m.p. 177–179; *R*_*f*_: 0.74; IR (KBr, *n*, cm^−1^):2873 (aliphatic H-C), 1656 (C=N of thiadiazole ring), 752 (S-C of thiadiazole ring), 1703 (C=O group), 1536 (aromatic C-C), 3061 (aromatic C-H), 732 (C-Cl of chlorobenzene); 1H NMR (400 MHz, d, ppm, DMSO-d6): 2.87 ppm (s, 3H, –CH_3_), 7.56 ppm (d, 2H, Ar-H), 7.97 ppm (d, 2H, Ar-H); ^13^C NMR (DMSO-d6): 13 ppm (CH_3_), 150, 162 ppm (thiadiazole), 190 ppm (C=O), 127, 127, 147, 134 ppm (aromatic).

#### 3.1.6. 5-Amino-1,3,4-Thiadiazol-2-yl Propanethioate (CPD-VI)

Yield: 0.87 g (46%); m.p. 231–233; *R*_*f*_: 0.64; IR (KBr, *n*, cm^−1^): 3319–3108 (N-H), 1661 (C=N of thiadiazole ring), 756 (S-C of thiadiazole ring), 1714 (C=O group), 2957 (aliphatic C-H); 1H NMR (400 MHz, d, ppm, DMSO-d6): 6.47 ppm (s, 2H, NH_2_), 1.14 ppm (t, 3H –CH_3_), 2.42 ppm (q, 2H, –CH_2_); ^13^C NMR (DMSO-d6): 159, 162 ppm (thiadiazole ring), 169 (C=O), 42 ppm (C-C=O), 42 ppm (C-R).

#### 3.1.7. 5-Amino-1,3,4-Thiadiazol-2-yl 4-Methoxybenzothioate (CPD-VII)

Yield: 1.58 g (59%); m.p. 233–235; *R*_*f*_: 0.62; IR (KBr, *n*, cm^−1^): 3327–3122 (N-H), 1668 (C=N of thiadiazole ring), 760 (S-C of thiadiazole ring), 1722 (C=O group), 1537 (aromatic C-C), 3052 (aromatic C-H), 1149 (methoxy O-C group); 1H NMR (400 MHz, d, ppm, DMSO-d6): 3.89 ppm (s, 2H, Ar-NH_2_), 3.29 ppm (s, 3H, –O–CH_3_), 7.01 ppm (d, 2H, Ar-H), 7.07 ppm (d, 2H, Ar-H); ^13^C NMR (DMSO-d6): 160, 163 ppm (thiadiazole ring), 192 ppm (C=O), 125, 128, 149, 158 ppm (aromatic), 67 ppm (O-C).

### 3.2. Biological Activity

#### 3.2.1. *In Vivo* Acute Toxicity Test

The toxicity study showed that all the animals administered with the test compounds were found to be safe at a dose of 550 mg/kg body weight. This was confirmed by lack of tremor, weight loss, paralysis, or aversive behaviors within 48 hours as well as in the next 14 days. There was no sign of diarrhea and deaths encountered with the treatment of the limit dose of all compounds, indicating that the median lethal oral dose of the compounds is greater than 550 mg/kg.

#### 3.2.2. Diuretic Activity: Effect on Urine Volume

1, 3, 4-Thiadiazole, the heterocyclic nucleus of present work, is a versatile pharmacophore, which exhibits a wide variety of biological activities. Different derivatives of the nucleus have tested for their diuretic activity and showed positive results [24, 28, 33]. In the present study, the synthesized compounds were evaluated for their diuretic activity, and their *in vivo* diuretic activity of the compounds is summarized in Table 2. Groups administered with CPD-I, CPD-II, CPD-IV, and CPD-V manifested pronounced rise in the cumulative urine output. All compounds increased urine volume starting from first hour of urine collection but significant increase in urine output was recorded in second hour of urine collection in CPD-І and CPD-VII (477%, *p* < 0.01) and (385%, *p* < 0.05), respectively, when compared to the negative control group. At third hour of urine collection, six compounds CPD-I (167%, *p* < 0.01), CPD-II (167%, *p* < 0.01), CPD-IV (194%,*p* < 0.001), CPD-V (118%, *p* < 0.01), CPD-VI (118%,*p* < 0.05) and CPD-VII (158%, *p* < 0.01) showed significant increase in urine volume when compared to the control group. CPD-I, CPD-II, CPD-IV, and CPD-V kept significant diuresis along fourth and fifth hours of diuresis. At twenty-fourth hour of diuresis, CPD-I (79%, *p* < 0.001), CPD-II (71%, *p* < 0.01), CPD-IV (108%, *p* < 0.001), CPD-V (88%, *p* < 0.001), and CPD-VII (55%, *p* < 0.05) resulted in significant diuresis when compared to the negative control group.

The urine output was increased by all the compounds even though the difference was insignificant in CPD-III and CPD-VI when it was compared to the control group. The highest diuresis was induced by CPD-IV and CPD-V, and generally, 5-methyl-substituted compounds showed significant increase in diuresis when they were compared to both the negative control group and 5-amino-substituted compounds. The steric bulk and the high electron density of substituents at 5^th^ position of 1, 3, 4-thiadiazoles are factors identified by 3D-QSAR comparative molecular field analysis to decrease diuretic activity of the compounds [31]. The low electron density of methyl group compared to the amino group at 5^th^ position might be the factor of increased diuretic activity of the 5-methyl-substituted 1, 3, 4-thiadiazoles. In another study, the diuretic activity of 1, 3, 4-thiadiazole derivatives decreases as the benzene ring at 5^th^ position has additional substituents on it [28]. Other similar studies showed that methyl substituents in 5^th^ position of 1, 3, 4-thiadiazoles showed significant diuresis in rats [34].

N-substitutions decrease the inhibitory activity of 1, 3, 4-thiadiazoles on CA-II isoenzyme, which is responsible target and the best candidate for diuretic compounds. As N-substituents get bulky, the inhibitory activity diminishes [35]. Hailu and Engidawork stated two hypothesis toward low inhibition of CA by 1, 3, 4-thiadiazole derivatives [36]. The first hypothesis was the bulk and electron density of substituents at 5^th^ position, and the second was the lack of additional hydrophobic moieties. In the case of present study also, it is possible to say the high electron density of amine substituents relative to the methyl group might be the reasons behind their relative better diuretic activity. The hydrophobic nature of methyl group also supports the hypothesis.

Furosemide showed significant diuresis from the very first hour of urine collection when compared to the control group as well as the test compounds in contrast to the significant diuresis recorded from the test compounds most of which started in the third hour of urine collection. The difference in onset of diuresis could be due to the difference in onset of action among different classes of diuretics. The onset of action of furosemide, which is loop diuretic, is within one hour of oral administration. On the other hand, carbonic anhydrase inhibitor class has onset of action about 2–3 hours with peak of action at 6–8 hours [29]. The test compounds of this project have claim of diuretic property by inhibition of carbonic anhydrase enzyme, and the slow onset of action is might be due to the same reason with other carbonic anhydrase inhibitors.

The calculated diuretic excretion, diuretic action, and diuretic activity of the test compounds are stated in Table 3. Diuretic activity is considered to be good if it is more than 1.50, moderate if it is between 1.00 and 1.50, mild if it is between 0.72 and 0.99, and nil if it less than 0.72 [36]. Accordingly, CPD-IV, CPD-II, CPD-I, and CPD-V showed mild diuretic activity and the rest of the compounds did not show diuretic activity with respect to furosemide. However, the compounds even might have better diuretic activity if they were compared with less potent diuretic drugs since furosemide is a high-ceiling and the most potent diuretic agent.

#### 3.2.3. Saluretic Activity: Effect on Electrolyte Content of the Urine

Electrolyte excretion is as important as excretion of water for diuretics to treat edema and ascites [30]. A compound to have a diuretic activity should result in increased urinary excretion of both water and electrolytes from the body. The increased diuresis by the test compounds of current study was also supported by increased electrolyte excretion. The cumulative urine samples collected in 24 hours were analyzed for the electrolyte content (Na^+^, K^+^, and Cl^−^) and presented in Table 4. All compounds increased the urinary Na^+^ excretion, but the maximum urinary Na^+^ excretion was recorded for CPD-IV (91%, *p* < 0.001) when compared to the control group. CPD-I (65%, *p* < 0.05), CPD-II (75%, *p* < 0.01), and CPD-V (64%, 0.05) also showed significant and urinary Na^+^ excretion when compared to the control group. The maximum K^+^ excretion was recorded for CPD-II (82%, *p* < 0.05), CPD-V (82%, *p* < 0.05), and CPD-IV (72%, *p* < 0.05) when compared to the control group. In the case of chloride ion excretion, although all compounds showed increased urinary excretion of Cl^−^ when compared to the control group, the difference was not as significant as Na^+^ and K^+^ excretion.

The saluretic index of Na^+^ of CPD-IV is higher than the rest of compounds and comparable with that of Fr10 (1.91 vs 1.97), while CPD-VI has the least saluretic index of Na^+^ (1.37). CPD-II and CPD-V showed highest saluretic indices of K^+^ (1.82) followed by CPD-IV (1.77), while Fr10 showed (2.05). CPD-V showed the highest value (1.65) of saluretic index of Cl^−^ and compounds CPD-II (1.59), CPD-IV (1.57), and CPD-I (1.54) showed high saluretic indices of Cl^−^, while Fr10 showed 2.67.

In addition, the calculated Na^+^/K^+^ of all the compounds is comparative to that of Fr10 and some of them showed slight increment. The calculated ratio is used to find aldosterone secretory index (natriuretic activity). If the calculated value is greater than 2.0, the compound will have favorable natriuretic effect, whereas if it is greater than 10.0, the compound will have a potassium-sparing effect [30]. However, none of the compounds increased the Na^+^/K^+^ ratio. Since it is clearly stated that potassium sparing diuretics usually increase the urinary Na^+^/K^+^ ratio, observations from current study suggest that the compounds do not have both natriuretic and potassium sparing effect since the maximum natriuretic activity recorded was 1.13 from CPD-I. In the case of Na^+^ and Cl^−^ excretion, all compounds showed increased excretion of both ions and CPD-I, CPD-II, CPD-IV, and CPD-V showed significant saluretic effect when compared with the control group. This is highly beneficial effect for removal of fluid from the body indifferent edematous conditions [30].

The CA enzyme inhibition of diuretic drugs can be estimated by calculated ratio of Cl^−^/Na^+^+K^+^. If the value is between 0.8 and 1.0, the drug or test compound has no CA inhibitory activity. The lower the ratio is observed, the stronger the CA inhibition is and vice versa [35]. All the test compounds had the ratio of Cl^−^/Na^+^+K^+^ below 0.8. The maximum ratio was observed for CPD-I (0.46), and the minimum was for CPD-IV (0.38). Therefore, it is reasonable to suggest that the possible mechanism of action of the test compounds is CA inhibition. In the case of CA inhibition, due to a large reabsorption capacity of Cl^−^ the expected rise in urinary excretion of Cl^−^ is smaller than the rise seen in other electrolytes [37]. The slight rise of Cl^−^ in this study could support the compounds have CA enzyme inhibition activity. The increased in alkalinity of urine is also one of the possible effect resulted from inhibition CA enzyme.

**Figure 1:**
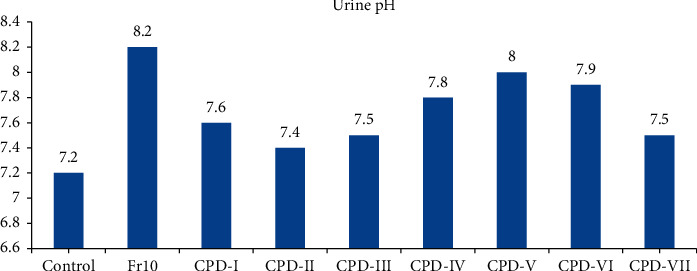
The effects of the test compounds on urine pH.

The pH of urine of all treatment groups, control group, and standard were measured from the fresh urine and the recorded results showed alkaline urine (Figure 1). From treatment groups, the maximum pH was recorded from CPD-V [8] and the least pH was from CPD-II (7.4). The results from test groups showed that an increased pH of urine than the control group. Compounds that act on the collecting tubule could increase urinary pH [30]. So, the rise in urinary pH could be due to either potassium sparing property or CA enzyme inhibition. However, none of the compounds showed potassium sparing property and this ruled out the possibility of having similar mechanism to that of the potassium sparing diuretics. It is known long ago that CA inhibitor diuretics such as acetazolamide, methazolamide, and other synthetic chemical compounds with 1, 3, 4-thiadiazole nuclease have CA inhibitory action. Thus, low value of Cl^−^/Na^+^+K^+^ ratio, the slight rise in Cl^−^ excretion, rise in urinary pH, and the known mechanism of action of compounds from this class support the notion that CA inhibition could be the possible mechanism of action of the test compounds.

To sum up, the findings of this study support synthesis of 2- and 5-thio-substituted 1, 3, 4-thiadiazoles is achieved by second-order nucleophilic substitution reaction using anhydrous salt and polar aprotic solvent media. The compounds showed diuretic activity in test animals by increasing volume of urine excretion as well as electrolytes. Different findings supported the compounds might have the CA inhibitory effect.

## 4. Conclusion

Seven new 1, 3, 4-thiadiazole derivatives were synthesized by second-order substitution reaction with high purity and good yield. The chemical structures of the compounds were confirmed by IR, ^1^H NMR, and ^13^C NMR spectroscopy. The safety profile of the compounds at test doses was established, and all the compounds did not cause recorded morbidity and mortality at test doses. The results from compounds provided evidence for 1, 3, 4-thiadiazole derivatives have in *vivo* diuretic activity. Increased excretion of urine volume and urinary electrolytes was observed after administration of the compounds. Generally, better diuresis was recorded for 5-methyl-substituted 1, 3, 4-thiadiazole-2-thiol derivatives than 5-amino-substituted ones.

## Figures and Tables

**Table 1:** The list of the synthesized compounds.

Compounds	*R*_1_	*R*_2_
CPD-I	CH_3_^−^	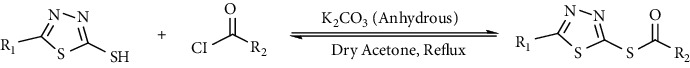
CPD-II	CH_3_^−^	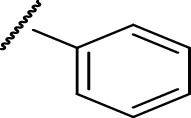
CPD-III	NH_2_^−^	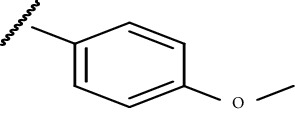
CPD-IV	CH_3_^−^	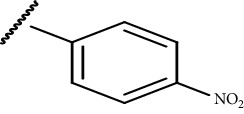
CPD-V	CH_3_^−^	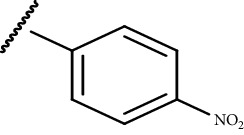
CPD-VI	NH_2_^−^	CH_3_CH_2_^−^
CPD-VII	NH_2_^−^	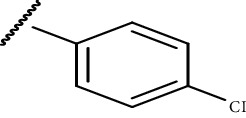

**Table 2:** The effect of the compounds on 24 hours of urine volume of mice.

Group	Volume of urine (ml)					
	1 hr	2 hr	3 hr	4 hr	5 hr	24 hr
Cont.	0.02 ± 0.01	0.13 ± 0.06	0.33 ± 0.04	0.37 ± 0.03	0.67 ± 0.06	1.13 ± 0.67
Fr10	1.12 ± 0.14^a3c3d3e3f3g3h3i3^	1.48 ± 0.14^a3c3d3e3f3g3h3i3^	1.63 ± 0.14^a3c3d3e3f3g3h3i3^	1.73 ± 0.14^a3c3d3e3f3g3h3i3^	1.90 ± 0.17^a3c2d2e3f2g3h3i3^	2.77 ± 0.14^a3c2d2e3h3i3^
CPD-І	0.37 ± 0.12	0.75 ± 0.09^a2e1^	0.88 ± 0.16^a2e1^	0.97 ± 0.04^a3e2^	1.27 ± 0.06^a2e2^	2.02 ± 0.10^a3h1^
CPD-II	0.33 ± 0.06	0.48 ± 0.12	0.88 ± 0.06^a2e1^	1.0 ± 0.06^a3e2^	1.23 ± 0.08^a2e2^	1.93 ± 0.08^a2^
CPD-III	0.08 ± 0.04	0.20 ± 0.08	0.27 ± 0.09	0.47 ± 0.07	0.57 ± 0.09	1.50 ± 0.03
CPD-IV	0.18 ± 0.04	0.40 ± 0.05	0.97 ± 0.07^a3e3^	1.12 ± 0.05^a3e3^	1.32 ± 0.09^a2e1^	2.35 ± 0.11^a3e2h3i1^
CPD-V	0.03 ± 0.03	0.30 ± 0.05	0.78 ± 0.05^a2e2^	0.95 ± 0.07^a3e2^	1.13 ± 0.13^e2^	2.13 ± 0.12^a3e1h2^
CPD-VI	0.27 ± 0.09	0.55 ± 0.12	0.72 ± 0.07^a3e2^	0.82 ± 0.09^a1^	0.87 ± 0.09	1.42 ± 0.11
CPD-VII	0.30 ± 0.01	0.63 ± 0.16^a1^	0.85 ± 0.10^a2e1^	0.90 ± 0.13^a2e1^	1.03 ± 0.10	1.75 ± 0.26^a1^

Values are expressed as mean ± S.E.M (*n* = 6); analysis was performed with one-way ANOVA followed by Tukey test; ^a^against control, ^b^against standard drug, ^c^against CPD-І, ^d^against CPD-II, ^e^against CPD-III; ^f^against CPD-IV; ^g^against CPD-V; ^h^against CPD-VI; ^i^against CPD-VII;^1^*p* < 0.05, ^2^*p* < 0.01, ^3^*p* < 0.001; cont: 2% Tween-80 in water; Fr10: furosemide 10 mg/kg; CPD-I: S-5-methyl-1, 3, 4-thiadiazol-2-yl 4-chlorobenzothioate; CPD-II: S-5-methyl-1, 3, 4-thiadiazol-2-yl 4-methoxybenzothioate; CPD-III: S-5-amino-1, 3, 4-thiadiazol-2-yl 4-nitrobenzothioate; CPD-IV: S-5-methyl-1, 3, 4-thiadiazol-2-yl 4-nitrobenzothioate; CPD-V: S-5-methyl-1, 3, 4-thiadiazol-2-yl 4-chlorobenzothioate; CPD-VI: S-5-amino-1, 3, 4-thiadiazol-2-yl propanethioate; CPD-VII: S-5-amino-1, 3, 4-thiadiazol-2-yl 4-methoxybenzothioate.

**Table 3:** Calculated urinary excretion, diuretic action, and activity of the test compounds.

Group	Urinary excretion (%)	Diuretic action	Diuretic activity
Cont.	116.7	1	
Fr10	316.7	2.71	1
CPD-І	238.8	2.05	0.75
CPD-II	253.3	2.17	0.80
CPD-III	187.3	1.60	0.59
CPD-IV	260.6	2.23	0.82
CPD-V	232.7	1.99	0.73
CPD-VI	182.1	1.56	0.56
CPD-VII	192.3	1.65	0.61

**Table 4:** The effect of the compounds on 24 hours of urine electrolyte excretion of mice.

Group	Urinary electrolyte excretion (mmol/L)			Saluretic index			Na^+^/K^+^	Cl^−^/Na^+^+K^+^
	Na^+^	K^+^	Cl^−^	Na^+^	K^+^	Cl^−^		
Cont.	51.17 ± 4.61	56.50 ± 6.20	47.91 ± 5.63				0.9	0.44
Fr10	100.67 ± 5.00^a3e1^	116.26 ± 16.54^a3d1e1i1^	128.03 ± 5.37^a3c3d3e3f3g3h3i3^	1.97	2.06	2.67	0.87	0.59
CPD-І	84.50 ± 8.20^a1^	74.56 ± 8.32	73.95 ± 3.55^a1^	1.65	1.32	1.54	1.13	0.46
CPD-II	89.67 ± 8.04^a2^	102.69 ± 12.73^a1^	76.38 ± 4.46^a2^	1.75	1.82	1.59	0.87	0.40
CPD-III	78.17 ± 6.38	72.05 ± 6.42	66.98 ± 4.94	1.53	1.28	1.39	1.08	0.45
CPD-IV	97.83 ± 3.35^a3^	99.80 ± 6.11^a1^	75.38 ± 5.97^a2^	1.91	1.77	1.57	0.98	0.38
CPD-V	83.83 ± 8.83^a1^	102.66 ± 8.95^a1^	79.15 ± 4.85^a2^	1.67	1.82	1.65	0.82	0.42
CPD-VI	70.50 ± 5.87	67.18 ± 4.98	61.91 ± 4.68	1.37	1.19	1.29	1.04	0.45
CPD-VII	78.33 ± 6.58	70.60 ± 4.44	65.11 ± 3.77	1.53	1.25	1.36	1.11	0.44

Values are expressed as mean ± S.E.M (*n* = 6); analysis was performed with one-way ANOVA followed by Tukey test; ^a^against control, ^b^against standard drug, ^c^against CPD-І, ^d^against CPD-II, ^e^against CPD-III; ^f^against CPD-IV; ^g^against CPD-V; ^h^against CPD-VI; ^i^against CPD-VII;^1^*p* < 0.05, ^2^*p* < 0.01, ^3^*p* < 0.001; cont: 2% Tween-80 in water; Fr10: furosemide 10 mg/kg; CPD-I: S-5-methyl-1, 3, 4-thiadiazol-2-yl 4-chlorobenzothioate; CPD-II: S-5-methyl-1, 3, 4-thiadiazol-2-yl 4-methoxybenzothioate; CPD-III: S-5-amino-1, 3, 4-thiadiazol-2-yl 4-nitrobenzothioate; CPD-IV: S-5-methyl-1, 3, 4-thiadiazol-2-yl 4-nitrobenzothioate; CPD-V: S-5-methyl-1, 3, 4-thiadiazol-2-yl 4-chlorobenzothioate; CPD-VI: S-5-amino-1, 3, 4-thiadiazol-2-yl propanethioate; CPD-VII: S-5-amino-1, 3, 4-thiadiazol-2-yl 4-methoxybenzothioate.

## Data Availability

The data are included in the text.
